# Elevated Cerebrospinal Fluid Ubiquitin Carboxyl‐Terminal Hydrolase Isozyme L1 in Asymptomatic *C9orf72* Hexanucleotide Repeat Expansion Carriers

**DOI:** 10.1002/ana.27133

**Published:** 2024-11-16

**Authors:** Elizabeth R. Dellar, Iolanda Vendrell, Benazir Amein, David G. Lester, Evan C. Edmond, Katie Yoganathan, Thanuja Dharmadasa, Aitana Sogorb‐Esteve, Roman Fischer, Kevin Talbot, Jonathan D. Rohrer, Martin R. Turner, Alexander G. Thompson

**Affiliations:** ^1^ Nuffield Department of Clinical Neurosciences University of Oxford Oxford UK; ^2^ Target Discovery Institute Centre for Medicines Discovery, Nuffield Department of Medicine University of Oxford Oxford UK; ^3^ Chinese Academy of Medical Science Oxford Institute University of Oxford Oxford UK; ^4^ The Florey Institute of Neuroscience and Mental Health University of Melbourne Parkville, Victoria Australia; ^5^ Dementia Research Centre, Department of Neurodegenerative Disease, UCL Queen Square Institute of Neurology University College London London UK; ^6^ UK Dementia Research Institute at University College London London UK; ^7^ Kavli Institute for Nanoscience Discovery University of Oxford Oxford UK

## Abstract

**Objective:**

To identify biochemical changes in individuals at higher risk of developing amyotrophic lateral sclerosis (ALS) or frontotemporal dementia (FTD) via *C9orf72* hexanucleotide repeat expansion (HRE) heterozygosity.

**Methods:**

Cross‐sectional observational study of 48 asymptomatic *C9orf72* HRE carriers, 39 asymptomatic non‐carrier controls, 19 people with sporadic ALS, 10 with *C9orf72* ALS, 14 with sporadic FTD, and 10 with *C9orf72* FTD. Relative abundance of 30 pre‐defined cerebrospinal fluid biomarkers of ALS and FTD were compared in asymptomatic *C9orf72* HRE carriers and age‐matched non‐carrier controls. Differential abundance of these proteins was quantified using data independent acquisition mass spectrometry or electro chemiluminescent assay for neurofilament light chain. Unbiased analysis of the entire cerebrospinal fluid proteome was then carried out.

**Results:**

Ubiquitin carboxyl‐hydrolase isozyme L1 levels were higher in asymptomatic *C9orf72* HRE carriers compared with age‐matched non‐carriers (log_2_fold change 0.20, FDR‐adjusted *p*‐value = 0.034), whereas neurofilament light chain levels did not significantly differ. Ubiquitin carboxyl‐hydrolase isozyme L1 levels remained elevated after matching of groups by neurofilament levels (*p* = 0.011), and after adjusting for age, sex, and neurofilament levels. A significant difference was also observed when restricting analysis to younger participants (<37) matched by neurofilament level (*p* = 0.007).

**Interpretation:**

Elevated cerebrospinal fluid ubiquitin carboxyl‐hydrolase isozyme L1 levels in *C9orf72* HRE carriers can occur in the absence of increased neurofilament levels, potentially reflecting either compensatory or pathogenic mechanisms preceding rapid neuronal loss. This brings forward the window on changes associated with the *C9orf72* HRE carrier state, with potential to inform understanding of penetrance and approaches to prevention. ANN NEUROL 2025;97:449–459

An intronic hexanucleotide repeat expansion (HRE) in *C9orf72* is the commonest monogenetic cause of the neurodegenerative diseases amyotrophic lateral sclerosis (ALS) and frontotemporal dementia (FTD), two clinically and pathologically overlapping syndromes.^1^, ^2^ The penetrance of *C9orf72* HRE is incomplete, meaning that not all carriers will develop ALS or FTD.^3^ There is currently no accurate way to predict which carriers will develop symptomatic ALS or FTD, nor its timing if so.

The *C9orf72* HRE is present in carriers from conception, expressed in multiple tissues beyond the nervous system, with evidence of non‐ATG translation into dipeptide repeat proteins found in early adulthood.^4^, ^5^ Symptoms of ALS and FTD because of *C9orf72* HRE, however, do not typically occur until middle age, although the age of onset varies.^6^ Once initiated, the neurodegenerative phase involving neuronal loss often progresses rapidly, with an average survival from symptom onset of less than 3 years in people with ALS, and approximately 9 years in those with FTD.^7^

This rapid progression of neurodegeneration following decades of normal functioning implies that age‐dependent failure of homeostatic mechanisms, which may involve incremental acquisition of toxicity, is necessary to initiate the development of symptomatic ALS or FTD in *C9orf72* HRE carriers. As for apparently sporadic cases of ALS, the relationship between age and incidence in *C9orf72*‐associated ALS fits with a notional concept of multiple discrete “steps” in pathogenesis.^8^

Because offering genetic testing for the monogenetic causes of ALS and FTD in affected individuals after diagnosis has become part of routine care, the healthy relatives of index cases increasingly become aware of the potential for a higher personal lifetime risk through their carrier status.^9^ Developing preventative treatment strategies is, therefore, an important goal. Major obstacles to this development include a lack of understanding of the biochemical underpinnings of both homeostasis and early disease mechanisms. There is a need for biomarkers that reflect these processes, and which can be used to both target only those at highest risk of phenoconversion and to demonstrate neuroprotection over what may need to be lifelong therapy.^10^

Neurofilament light chain (NFL) is a measure of the rate of axonal degeneration. In carriers of ALS‐causing *SOD1* variants, it becomes elevated in blood 6 to 12 months before the development of symptomatic ALS.^11^ This latency may be longer for the *C9orf72* HRE and in relation to those who develop FTD rather than ALS (because of a slower disease course).^11^, ^12^ Biomarkers indicating cellular dysfunction or compensatory mechanisms before this phase of neurodegeneration associated with raised NFL are needed.

Proteomic analysis of cerebrospinal fluid (CSF) allows the simultaneous measurement of hundreds or thousands of proteins in the biofluid proximal to the cells affected by ALS and FTD.^13^ The aim of this study is to use proteomic analysis to identify biochemical changes in individuals at higher risk of developing ALS or FTD via *C9orf72* HRE heterozygosity, using both a candidate protein and unbiased approach.

## Methods

### Participants and Samples

Comparative proteomic analysis was undertaken in CSF samples taken from (1) asymptomatic carriers of *C9orf72* HRE, (2) asymptomatic non‐carriers, (3) people living with *C9orf72‐*related ALS or FTD, and (4) people living with apparently sporadic ALS or FTD.

Participants were of adult age. Symptomatic participants were recruited from specialized ALS and FTD clinics at Oxford University Hospitals National Health Service (NHS) Foundation Trust and University College London Hospitals, respectively. At‐risk first‐degree relatives of affected individuals with a confirmed *C9orf72* HRE were offered participation in the study via their affected relative. Asymptomatic non‐carrier participants were typically friends or spouses (non‐relatives) of people living with ALS or FTD, or of their first‐degree relatives, but without a personal family history of ALS or FTD.

At‐risk participants underwent testing for *C9orf72* HRE through a double blinded testing protocol using repeat primed polymerase chain reaction (RP‐PCR) undertaken using clinically validated methods by the Clinical Genetics Laboratory, Oxford University Hospitals NHS Foundation Trust, or University College London Hospital NHS Foundation Trust. Results were not fed back to participants, but matched to participant data and identifiers removed via an independent genetic guardian to allow analysis. Access to unblinded genetic status and data fields that could potentially be used to identify individual study participants (eg, age and sex) was limited to non‐participant facing members of the research team to negate the risk of inadvertent disclosure. Asymptomatic at‐risk family members found not to be carrying a *C9orf72* HRE following blinded genetic testing were analyzed as part of the asymptomatic non‐carrier participant group. Clinical data was ascertained on the day of lumbar puncture. Participants underwent structured physical examination and cognitive examination using the Edinburgh cognitive and behavioral ALS screen (ECAS) (Oxford) or Clinical Dementia Rating Dementia Staging Instrument (CDR) plus National Alzheimer's Coordinating Center Behavior and Language Domains (NACC FTLD) (University College London). *C9orf72* HRE carriers were classified as asymptomatic if they scored 0 on the CDR plus NACC FTLD global score, or symptomatic if ≥1 (University College London) or ECAS total score ≤105 (Oxford).^14^, ^15^

Studies were conducted under United Kingdom (UK) Health Research Authority approvals (research ethics committee references 16/SC/0277 and 14/0377). Participants gave informed consent, and for those individuals lacking mental capacity, advice of a consultee was sought.

CSF was obtained by lumbar puncture using standard clinical procedures. Samples were centrifuged at 2,300*g* for 10 minutes at 4°C, or at 1,750*g* for 5 minutes at 22°C, then transferred to labelled polypropylene cryostorage tubes and stored at −80°C. All samples are processed within 2 hours from extraction.

### Sample Preparation

Samples of CSF were thawed on ice and 50μL mixed 1:1 with solubilization buffer (10% sodium dodecyl sulfate in 100mM triethylammonium bicarbonate [TEAB]). Ten samples were prepared in duplicate for quality control purposes (independent digestions) and distributed randomly across plates. Lysates were reduced with dithiothreitol (final concentration 5mM) and incubated for 30 minutes at room temperature. Samples were then alkylated with iodoacetamide (final concentration 20mM) and incubated for 30 minutes at room temperature in the dark. Samples were acidified with phosphoric acid (final concentration 2.5%) before addition of six volumes of binding buffer (90% aqueous methanol with 100mM TEAB). Lysates were then applied to wells of 96‐well S‐Trap plate (Protifi, Fairport, NY) and centrifuged at 2,000*g*. This was repeated until all samples had been passed through. Bound protein was washed three times with 200μL of binding buffer. A total of 125μL of enzyme solution (1.8μg trypsin/Lys‐C mix in 50mM TEAB) was added to each well of the dried plate and incubated for 1 hour at 37°C in a water‐saturated atmosphere. A further 75μL of 50mM TEAB was then added to each well before overnight incubation. A final 75μL of 50mM TEAB was then added and the plate centrifuged at 2,000*g* to elute peptides. Further elution was carried out by addition of 80μL 0.2% aqueous formic acid and 80μL 50% aqueous acetonitrile with 0.2% formic acid. Peptides were frozen, then thawed to dry down and resuspend in 60μL 2% acetonitrile/0.1% formic acid before analysis.

### Mass Spectrometry

Peptides were analyzed by liquid chromatography–tandem mass spectrometry (LC–MS/MS) with an UltiMate 3000 HPLC coupled to an Orbitrap Ascend Tribrid instrument (ThermoFisher, Waltham, MA) using a nano‐EASY spray source. Tryptic peptides were loaded onto a AcclaimPepMap100 trap column (100μm × 2cm, PN164750; ThermoFisher) and separated on a 50cm EasySpray column (ES903, ThermoFisher) using a 60 minutes linear gradient from 2 to 35% acetonitrile, 0.1% formic acid and at 250nl/min flow rate. Both trap and column were kept at 50°C. MS data were acquired in data‐independent mode (DIA) with minor changes from previously described method.^13^, ^16^, ^17^ Briefly, MS1 scans were collected in the orbitrap at a resolving power of 45K at m/z 200 over m/z range of 350 to 1,650 m/z. The MS1 normalized automatic gain control was set at 125% (5e5ions) with a maximum injection time of 91ms and a radio frequency lens at 30%. DIA MS2 scans were then acquired using the tMSn scan function at 30K orbitrap resolution over 40 scan windows with variable width, with a normalized automatic gain control target of 1,000%, maximum injection time set to auto and a 30% collision energy.

Raw MS files were analyzed in a library‐free manner with DIA‐NN v8.^18^ Spectra were searched against the UniprotSwissprot reviewed human proteome (downloaded February 2022 containing 20,386 sequence), with match‐between runs, 1% peptide false discovery rate and allowing for 1 missed cleavage. N‐terminal excision and oxidation of methionine were included as variable modifications.

### NFL Measurement

CSF NFL was measured on the same samples after one additional freeze thaw, using the R‐plex NFL Meso Scale Discovery assay (Meso Scale Discovery, Rockville, MD) with duplicate samples at 1:2 dilution in diluent 12, according to the manufacturer's instructions. All samples were within assay working range of 12 to 50,000pg/ml. Inter‐assay coefficient of variation was 12.2% and mean intra‐assay coefficient of variation was 4.62%. One sample value was imputed according to the median value for its group.

### Statistical Analysis

Data were filtered to exclude protein groups that were present in <50% samples of any single group. Abundance values were log_2_ transformed and normalized using the median abundance of the 90% protein groups with the lowest variance and scaled by median absolute deviation in Python. Data was then imputed by multivariate feature imputation using an iterative method with Sci‐Kit Learn IterativeImputer to give a single output dataset (max iterations = 50, random state = 0).^19^ Quality control of samples was carried out by hierarchical clustering, with any samples clustering more closely than known duplicates excluded from further analysis (Fig. S1). To ensure appropriate age‐matching while maximizing statistical power, in each pairwise group analysis comparing ALS, FTD, and asymptomatic *C9or72* HRE carrier groups with asymptomatic non‐carrier controls, the maximal number of the 39 asymptomatic non‐carrier controls samples were included while meeting the condition that significance of the difference between mean group age was greater than 0.1. The distribution of imputed log‐transformed protein abundances was tested for normality with Shapiro–Wilk test on a protein‐by‐protein level, with 52% of proteins following a non‐normal distribution. Primary univariate analysis was first carried out on a set of 30 pre‐defined proteins of interest, previously shown to differ between people with ALS or FTD and controls or with relevance to specific prognostic factors from our previous work and published literature. A full list of target proteins is provided in (Table 1). NFL, measured using NFL electrochemiluminescence assay in the same CSF samples, was also included because this is an established marker of axonal degeneration in ALS and FTD and currently represents the earliest biochemical marker of an active pathological process in ALS and FTD gene carriers. Analysis was performed using Wilcoxon Rank sum test in R with Benjamini‐Hochberg method for false‐discovery rate (FDR) correction, and a cut‐off of FDR‐adjusted *p* < 0.05. Secondary univariate analysis was carried out on all proteins present in the filtered dataset, with an FDR‐cut‐off of 0.1.

**TABLE 1:** Preselected Target Proteins

Gene symbol	Gene name	Change	Reference
NEFL	Neurofilament light chain	↑ (pre)symptomatic ALS/FTD	^11^, ^12^
UCHL1	Ubiquitin carboxyl‐terminal hydrolase isozyme L1	↑ ALS	^13^, ^20^, ^21^
CHI3L1	Chitinase‐3‐like protein 1	↑ ALS	^22^, ^23^
CHI3L2	Chitinase‐3‐like protein 2	↑ ALS	^22^, ^23^
CHIT1	Chitotriosidase‐1	↑ ALS	^22^, ^23^
GPNMB	Transmembrane glycoprotein NMB	↑ ALS	^20^, ^21^
CD163	Scavenger receptor cysteine‐rich type 1 protein M130	Survival association	^13^ – Inflammatory module
CD44	CD44 antigen	–	^13^– Inflammatory module
PI16	Peptidase inhibitor 16	–	Unpublished analysis spinal vs bulbar onset ALS
FCGR3A	Low affinity immunoglobulin γ Fc region receptor III‐A	↑ ALS	^13^
LTBP2	Latent‐transforming growth factor β‐binding protein 2	Survival association	^13^– Inflammatory module; 50
PGRMC1	Membrane‐associated progesterone receptor component 1	DPR association	^13^ – ER module
SERPINA3	α‐1‐antichymotrypsin	↑ ALS	^13^, ^20^, ^21^
CFD	Complement factor D	↑ ALS	^13^
MB	Myoglobin	↑ ALS	^13^
VDAC1	Voltage dependent anion‐selective channel 1	↑ ALS	^13^
C1S	Complement C1s subcomponent	Survival association	^13^ – Inflammatory module
C1R	Complement C1r subcomponent	Survival association	^13^ – Inflammatory module
C7	Complement component C7	Survival association	^13^ – Inflammatory module
NCAM1	Neural cell adhesion molecule 1	DPR association	^13^ – ER module
PDIA3	Protein disulfide isomerase A3	DPR association	^13^
PDIA4	Protein disulfide isomerase A4	DPR association	^13^ – ER module
CALR	Calreticulin	DPR association	^13^ – ER module
HSPA5	Endoplasmic reticulum chaperone BiP	DPR association	^13^ – ER module
CLEC11A	C‐type lectin domain family 11 member A	DPR association	^13^ – ER module
COL1A1	Collagen α‐1(I) chain	–	Unpublished analysis spinal vs bulbar onset ALS; ^24^
SLC38A10	Solute carrier family 38 member 10	–	Unpublished analysis spinal vs bulbar onset ALS; ^25^
LTBP4	Latent‐transforming growth factor β‐binding protein 4	–	Unpublished analysis spinal vs bulbar onset ALS
NID1	Nidogen‐1	–	Unpublished analysis spinal vs bulbar onset ALS
NPTX2	Neuronal pentraxin‐2	↓ ALS/FTD	^20^, ^26^

Arrows indicate increase or decrease in ALS or FTD CSF. ALS = amyotrophic lateral sclerosis; DPR = disability progression rate; FTD = frontotemporal dementia.

Sensitivity analysis was carried out by: (1) matching groups by mean NFL levels to a *p*‐value >0.5 (therefore, excluding asymptomatic C9orf72 HRE carriers with high NFL), (2) using linear regression to adjust for age and sex, (3) using linear regression to adjust for NFL level and sex and (4) analyzing Oxford and University College London datasets separately. Cliff's delta effect sizes (δ) were calculated in R using effSize package.

## Results

Demographic information for the full cohort is provided in Table 2 and for individual contrasts in Table S1. A total of 1,336 protein groups were quantified, 753 (56%) of which were quantified in all samples and 1,013 (76%) of which were quantified in at least 90% of samples. The data were filtered by proteins present in at least 50% of samples in one of the six groups (sporadic ALS, *C9orf72* ALS, sporadic FTD, *C9orf72* FTD, asymptomatic *C9orf72* HRE carrier, and asymptomatic non‐carrier), leaving 1,245 for analysis.

**TABLE 2:** Participant Demographics

	Asymptomatic		ALS		FTD		Overall	
	*C9orf72* HRE carrier	Non‐carrier	*C9orf72* HRE carrier	Non‐carrier	*C9orf72* HRE carrier	Non‐carrier	*C9orf72* HRE carrier	Non‐carrier
No.	48	39	10	19	10	14	68	72
Female, n (%)	26 (54.2%)	21 (53.8%)	5 (50.0%)	5 (26.3%)	3 (30.0%)	2 (14.3%)	34 (50.0%)	28 (38.9%)
Age at sampling, median (IQR), yr	39.5 (33.0–45.7)	48.2 (37.2–55.6)	51.5 (49.8–56.2)	59.6 (56.0–69.4)	58.3 (56.8–68.6)	61.8 (59.3–67.1)	44.5 (35.8–55.7)	55.9 (45.7–63.0)
Age of symptom onset, median (IQR), yr	–	–	50.6 (47.4–54.9)	57.7 (51.5–67.5)	52.0 (43.0–55.0)	57.0 (56.0–62.3)	50.6 (46.6–55.2)	57.7 (54.2–64.9)

ALS = amyotrophic lateral sclerosis; ALSFRS‐R = revised ALS functional rating scale; FTD = frontotemporal dementia; HRE = hexanucleotide repeat expansion; IQR = interquartile range.

### Analysis of Preselected Target Proteins

The principal aim of this study was to identify evidence of compensatory protective or pathogenic mechanisms occurring in asymptomatic *C9orf72* HRE carriers. We profiled the protein content of CSF from all participant groups using MS with library‐free DIA acquisition. Primary analysis compared samples from asymptomatic *C9orf72* HRE carriers with age‐matched non‐carrier control participants, using a manually curated target list of 30 target proteins (Table 1). These proteins were selected before data analysis, based primarily on our previous work comparing the CSF proteome of participants with sporadic ALS with controls, but with additional proteins of interested such as Neuronal pentraxin‐2 (NPTX2) and transmembrane glycoprotein NMB (GPNMB) identified from the literature.^13^, ^20^, ^26^ Analysis of target proteins was also conducted comparing samples from people with *C9orf72* ALS, *C9orf72* FTD, sporadic ALS and sporadic FTD, and age‐matched asymptomatic non‐carrier participants.

**FIGURE 1:**
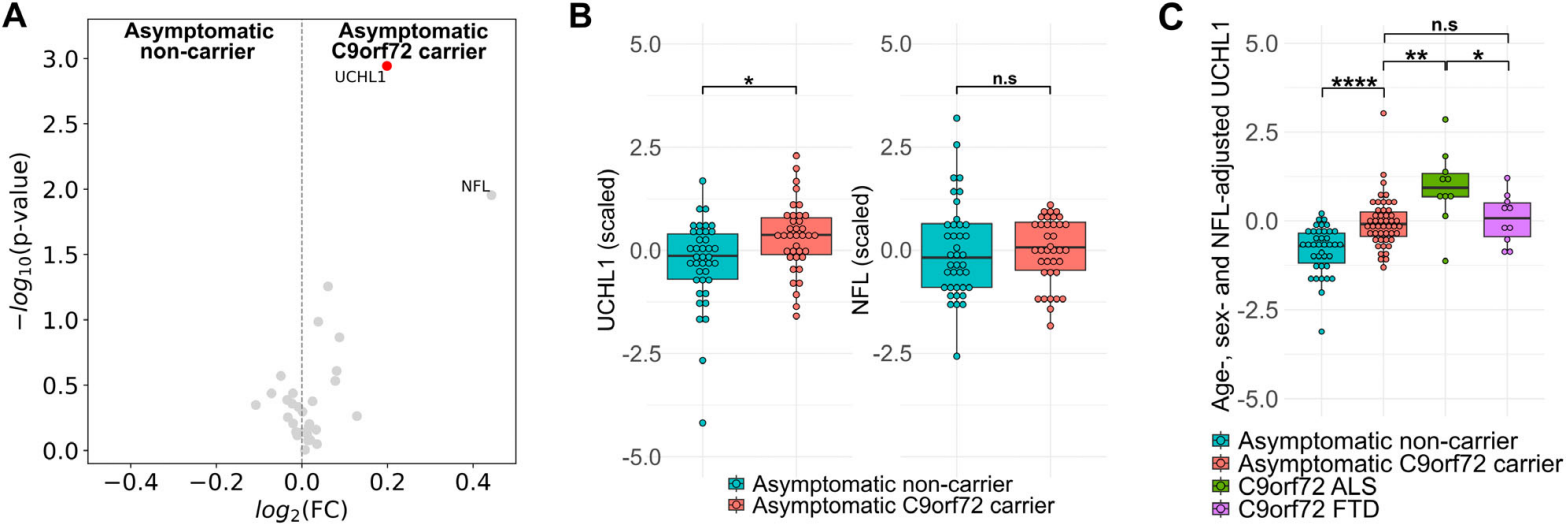
UCHL1 is increased in abundance in cerebrospinal fluid (CSF) of *C9orf72* HRE carriers. (A) Volcano plot showing log_2_ fold change versus raw *p*‐value for asymptomatic *C9orf72* HRE carriers compared to age‐matched (*p* > 0.1) non‐carrier controls for 30 preselected target proteins. Red points indicate proteins reaching statistical significance at FDR <0.05. NFL measured by electro chemiluminescent assay, all other proteins quantified by mass spectrometry. (B) Boxplots showing UCHL1 and NFL levels for asymptomatic *C9orf72* HRE carriers (n = 37) and non‐carrier controls (n = 39) after matching by mean NFL (*p* = 0.58). Values log‐transformed, centered to 0 and scaled to unit variance for comparability of effect sizes. (C) Boxplot showing UCHL1 levels for asymptomatic non‐carriers (n = 39), asymptomatic *C9orf72* HRE carriers (n = 43), *C9orf72* ALS (n = 10) and *C9orf72* FTD (n = 10) participants after correction for age, sex and NFL levels. Values log‐transformed, centered to 0 and scaled to unit variance for comparability of effect sizes. NFL measured by electro chemiluminescent assay, UCHL1 quantified by mass spectrometry. Significance tested by Wilcoxon Rank Sum test with FDR correction by Benjamini Hochberg procedure. *False discovery rate (FDR) <0.05, **FDR <0.01, ***FDR <0.001, ****FDR <0.0001. ALS, amyotrophic lateral sclerosis; FTD, frontotemporal dementia; HRE, hexanucleotide repeat expansion; NFL, neurofilament light chain; UCHL1, ubiquitin carboxyl‐terminal hydrolase isozyme L1. [Color figure can be viewed at www.annalsofneurology.org]

One protein was elevated in asymptomatic *C9orf72* HRE carriers after correcting for multiple comparisons: ubiquitin carboxyl terminal hydrolase L1 (UCHL1) (log_2_fold change 0.20, FDR‐adjusted *p*‐value = 0.034) (Fig 1A, B). CSF NFL did not differ significantly between asymptomatic *C9orf72* HRE carriers and age‐matched non‐carrier controls after FDR correction (fold change 0.44, FDR‐adjusted *p*‐value = 0.167) (Fig 1A). UCHL1 was also elevated in CSF in comparisons of all other symptomatic groups with age matched non‐carrier controls (sporadic ALS log_2_fold change 0.53, FDR‐adjusted *p*‐value <0.001; *C9orf72* ALS log_2_fold change 0.51, FDR‐adjusted *p*‐value <0.001; sporadic FTD log_2_fold change 0.41, FDR‐adjusted *p*‐value = 0.007; *C9orf72* FTD log_2_fold change 0.31, FDR‐adjusted *p*‐value = 0.010) (Fig S2).

We then carried out sensitivity analysis, stringently matching asymptomatic participant groups by mean NFL (*p* > 0.5). UCHL1 remained significantly elevated in asymptomatic *C9orf72* HRE carriers (*p* = 0.011) (Fig 1B). Adjusting for age, sex, and NFL level accentuated differences in UCHL1 levels between asymptomatic *C9orf72* HRE carriers compared to non‐carrier controls (*p* < 0.001) (Fig 1C). Adjusted UCHL1 levels remained higher in symptomatic *C9orf72* ALS compared to asymptomatic carriers (*p* = 0.008), but adjusted UCHL1 levels in *C9orf72* FTD were similar to those in asymptomatic carriers (*p* = 0.367). Furthermore, subgroup analysis by cohort demonstrated that UCHL1 levels were significantly higher in asymptomatic *C9orf72* HRE carriers in both Oxford (*p* = 0.005) and University College London cohorts (*p* = 0.016) (Fig S3). NFL levels in the Oxford cohort showed no difference between asymptomatic *C9orf72* HRE carriers and asymptomatic non‐carrier controls (*p* = 0.294) with a non‐significant increase in the University College London cohort (*p* = 0.061).

**FIGURE 2:**
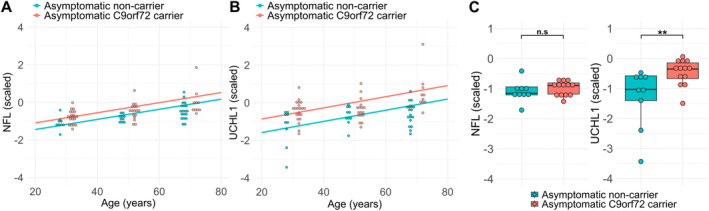
UCHL1 is increased in cerebrospinal fluid (CSF) of both young and older *C9orf72* HRE carriers. (A) Regression of NFL level against age in asymptomatic *C9orf72* HRE carriers and non‐carrier controls. (B) Regression of UCHL1 level against age in asymptomatic *C9orf72* HRE carriers and non‐carrier controls with age. Overlaid points binned by age (<37 years, 38–48 years, >48 years). Mean age within these age groupings did not differ significantly (*p* = 0.47, *p* = 0.78 and *p* = 0.41, respectively). (C) Boxplots showing UCHL1 and NFL levels in the youngest age group (<37 years) for asymptomatic *C9orf72* HRE carriers (n = 13) and non‐carrier controls (n = 9) after matching by mean NFL. Values log‐transformed, centered to 0 and scaled to unit variance for comparability of effect sizes. Significance tested by Wilcoxon Rank Sum test *p < 0.05; **p < 0.01; ***p < 0.001; ****p < 0.0001. HRE, hexanucleotide repeat expansion; NFL, neurofilament light chain; UCHL1, ubiquitin carboxyl‐terminal hydrolase isozyme L1. [Color figure can be viewed at www.annalsofneurology.org]

To explore the possibility that elevated UCHL1 levels reflected an active neurodegenerative process in asymptomatic *C9orf72* HRE carriers, we next aimed to assess the relationship with age and carrier status. In asymptomatic participants, both NFL and UCHL1 levels were associated with age (NFL gradient 0.03 standard deviations per year; *p* < 0.001; UCHL1 gradient 0.03 standard deviations per year; *p* < 0.001) and both showed a significant effect of *C9orf72* HRE carrier status (NFL 0.35 standard deviations difference, *p* < 0.001; UCHL1 0.72 standard deviations difference, *p* < 0.001) (Fig 2A, B). This result was maintained after exclusion of an outlying sample showing high levels of both NFL and UCHL1 (Fig S4).

We also stratified asymptomatic participants into age three groups (although ensuring at least two male and two female participants in each group and each age stratum to preserve blinded genetic status) to identify the age at which the elevation in UCHL1 could be detected relative to NFL. After adjustment for age, sex, and NFL levels, median UCHL1 levels were higher in young (<37), intermediate (37–48), and older (>48) age groups (effect size δ = 0.75 95% confidence interval [CI], 0.40–0.91, *p* = 0.0008; δ = 0.48 CI 0.04–0.76, *p* = 0.037; δ = 0.80 CI, 0.46–0.94, *p* = 0.0002, respectively). In the younger (<37 years) participants, UCHL1 levels again remained significant after stringent matching of groups by NFL level (δ = 0.60 CI, 0.20–0.83, *p* = 0.007) (Fig 2C).

Of the other 28 target proteins, 14 demonstrated altered abundance in at least one other contrast (Fig S2). The chitinase proteins (Chitotriosidase 1, Chitinase 3‐like protein 1, and Chitinase 3‐like protein 2), GPNMB, and CD44 were all significantly increased in abundance in both sporadic and *C9orf72* ALS, but only Chitinase 3‐like protein 1 was significantly raised in sporadic FTD. None were differentially abundant in *C9orf72* FTD compared with age‐matched controls. Additional inflammation‐associated proteins were raised in sporadic ALS (Scavenger receptor cysteine‐rich type 1 protein M130‐CD163, Complement component C7, and peptidase inhibitor 16), whereas a single protein (latent‐transforming growth factor β‐binding protein 4) was decreased in abundance in *C9orf72* ALS. Low affinity immunoglobulin γ Fc region receptor III‐A was increased in abundance in both sporadic and *C9orf72* FTD, in addition to CD163, α‐1‐antichymotrypsin (SERPINA3), and latent‐transforming growth factor β‐binding protein 2 in sporadic FTD only. Neuronal pentraxin‐2 was also found to be decreased in abundance in sporadic FTD.

### Analysis of the Unbiased CSF Proteome

**FIGURE 3:**
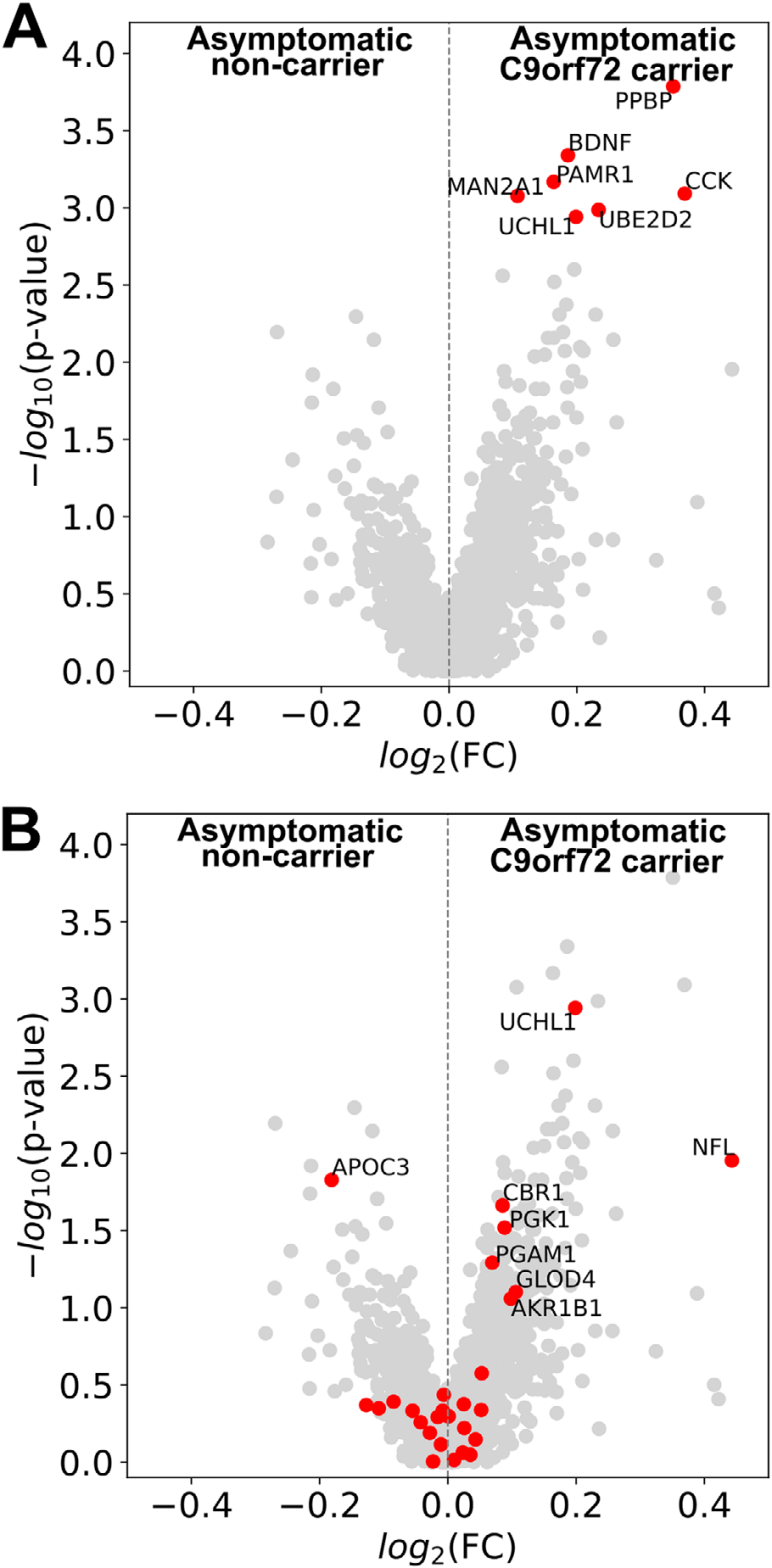
Unbiased cerebrospinal fluid (CSF) proteomic analysis in asymptomatic *C9orf72* ALS HRE carriers. (A) Volcano plot showing log_2_ fold change versus raw *p*‐value for asymptomatic *C9orf72* HRE carriers compared to age‐matched (*p* > 0.1) asymptomatic non‐carrier participants. Red points indicate proteins showing lower FDR‐adjusted *p*‐value than UCHL1. (B) Volcano plot showing log_2_ fold change versus raw *p*‐value for asymptomatic *C9orf72* HRE carriers compared to age‐matched (*p* > 0.1) asymptomatic non‐carrier participants. Red points indicate proteins that showed significant differences in other contrasts. NFL measured by electro chemiluminescent assay; all other proteins quantified by mass spectrometry. Significance tested by Wilcoxon Rank sum test with FDR correction by Benjamini Hochberg procedure. FC, fold change; FDR, false discovery rate adjusted *p*‐value; HRE, hexanucleotide repeat expansion; PPBP, pro‐platelet basic protein; BDNF, brain derived neurotrophic factor; PAMR1, peptidase domain containing associated with musicle regeneration 1; MAN2A1, mannosidase α class 2A member 2; CCK, cholecystokinin; UBE2D2, ubiqutin conjugating enzyme E2 D2; UCHL1, ubiquitin carboxyl‐terminal hydrolase isozyme L1; NFL, neurofilament light chain; APOC3, apolipoprotein C3; CBR1, carbonyl reductase 1;PGK1, phosphoglycerate kinase 1; PGAM1, phosphoglycerate mutase 1; GLOD4, glyoxalase domain containing 4; AKR1B1, aldo‐keto reductase family 1 member B. [Color figure can be viewed at www.annalsofneurology.org]

No differences between asymptomatic *C9orf72* HRE carriers and age matched non‐carrier controls were significant after FDR correction in the total dataset, although six proteins had a lower FDR than UCHL1: brain derived neurotrophic factor, peptidase domain containing associated with muscle regeneration 1, pro‐platelet basic protein, mannosidase α class 2A member 2, ubiquitin conjugating enzyme E2 D2, and cholecystokinin (Fig 3A).

We also carried out comparisons of sporadic ALS and FTD, and *C9orf72* ALS and FTD with age matched non‐carrier control participants in the full dataset. A total of 29 proteins showed differential abundance in one or more of these comparisons (FDR <0.1) (Fig S5). Restricting analysis of asymptomatic *C9orf72* HRE carriers versus non‐carrier controls to proteins differentially abundant between these other contrasts (ie, symptomatic disease vs asymptomatic non‐carriers) did not identify additional differentially abundant proteins after FDR correction (Fig 3B).

## Discussion

This study sought to identify and characterize alterations in the CSF proteome of asymptomatic carriers of *C9orf72* HRE. It identified elevation of both CSF UCHL1 and NFL in the asymptomatic *C9orf72* HRE carrier group, which was present in some asymptomatic carriers of a younger age (<37 years). Although elevation in plasma NFL has been documented in *C9orf72* carriers up to 30 years before the development of symptomatic disease,^12^ the pattern of raised UCHL1 in CSF observed here persisted even after accounting for NFL, including in younger participants. UCHL1 appears, therefore, to reflect an earlier biochemical milieu, occurring before the onset of overt neurodegeneration that characterizes the peri‐symptomatic phase of ALS and FTD.

Several small studies have found no difference in NFL levels in CSF in small mixed groups of asymptomatic *C9orf72* HRE, *TARDBP*, *GRN*, and *FUS* variant carriers (n = 12, Weydt et al^27^; n = 25, Meeter et al^28^; n = 8, Scherling et al^29^). However, in a large study of FTD variant carriers, increased NFL levels were found to be increased in plasma, with changes first evident up to 30 years before symptom onset in *C9orf72* FTD, 15 years prior in *GRN* FTD, but not in *MAPT* FTD.^12^ Our study shows a small increase in mean NFL in the asymptomatic group in a relatively large (n = 44) cohort of a single variant compared with similar studies. The principal limitations are its cross‐sectional nature, which precludes precisely defining the time course of elevation in individual participants. However, at present there is only a small group of asymptomatic carriers who have been studied through to phenoconversion, and it will be necessary to build larger cohorts through international collaboration to study the time course of this change.

Elevation of CSF UCHL1 in people with ALS has been robustly demonstrated by independent groups using multiple orthogonal methods.^13^, ^20^, ^21^, ^30^, ^31^, ^32^, ^33^ CSF UCHL1 level correlates with disability progression rate and overall survival in ALS.^13^, ^31^ Two studies have also shown that UCHL1 is increased in plasma or serum in ALS patients compared to healthy participants (but not disease controls), with one demonstrating that UCHL1 levels gave additional separation of survival in patients with low NFL.^30^, ^33^ UCHL1 levels were previously shown to correlate with phosphorylated neurofilament heavy chain in ALS CSF (r = 0.49),^13^ and we observed a stronger relationship with NFL (r = 0.74 across all groups) in the current study. We also showed that UCHL1 is raised in both apparently sporadic and *C9orf72‐*related FTD, indicating that this elevation is neither ALS‐ nor *C9orf72‐*specific.

Our study demonstrates that elevation of UCHL1 in CSF is detectable in asymptomatic *C9orf72* HRE carriers, even in the absence of raised levels of NFL, indicating that it may be an earlier event. Two previous studies of the CSF proteome in asymptomatic carriers of ALS‐causing genetic variants including *C9orf72* HRE did not identify significant elevation of UCHL1.^20^, ^32^ The penetrance of *C9orf72* HRE is incomplete, so each cohort studied likely reflects participants of highly variable proximity to any phenoconversion. Hence, the smaller number of carriers studied or, in one study, the amalgamation of carriers of multiple causative variants into a single group, may explain the lack of difference previously observed.^20^, ^32^ The use of an ionic surfactant‐based sample preparation method in this study may also have led to better detection or more accurate quantification of membrane‐bound proteins such as UCHL1, which has both a free form and a membrane‐associated form, the latter of which has been reported to be of greater proportional abundance in neurons.^34^ Establishing which form of UCHL1 is increased in the CSF will be necessary to determine appropriate sample preparation conditions for compatibility in orthogonal immunoassay methods. Much further work to establish reliable quantitative assays of UCHL1 in CSF and blood is necessary before translation.

UCHL1 is expressed abundantly throughout the neuronal soma, axonal, and synaptic compartments.^35^, ^36^, ^37^ It is expressed highly in Betz cells of the primary motor cortex, as well as other upper motor neurons and a subset of lower motor neurons.^38^, ^39^ UCHL1 has been proposed to play a role in the maintenance of the free ubiquitin pool, preservation of axonal integrity, and synaptic remodelling.^35^, ^37^ Although relatively restricted to the central nervous system, UCHL1 may also be expressed in microglia and other immune cells and play a role in regulating inflammasome formation.^37^, ^40^, ^41^

UCHL1 has been implicated in a range of neurodegenerative conditions. A homozygous loss of function mutation results in early onset progressive disorder with cerebellar ataxia with upper motor neuron dysfunction.^42^ Heterozygous loss‐of‐function variants in UCHL1 lead to a neurodegenerative disease with pyramidal features, ataxia, optic, and peripheral neuropathy.^43^ Decreased abundance of UCHL1 has been reported in CSF from patients with synucleinopathies, and in tissue from FTD, people with Alzheimer's disease and Parkinson's disease.^44^, ^45^, ^46^, ^47^ Conversely, in the membrane‐specific fraction of postmortem frontal cortex in Alzheimer cases, increased UCHL1 has been observed.^48^

UCHL1 knockout mice develop degeneration of corticospinal tract pyramidal cells, which show increased susceptibility to endoplasmic reticulum stress.^38^ Viral vector‐mediated delivery of the UCHL1 gene to these cells improved cytoarchitectural integrity, and reduced protein aggregation in both human *SOD1* ^G93A^ and *TARDBP* ^A315T^ mouse models.^49^ An earlier study also observed that the subset of UCHL1‐eGFP‐labelled lower motor neurons were more resistant to degeneration in the human *SOD1* ^G93A^ mouse.^39^ In a mouse model of the lower motor neuronopathy spinal muscular atrophy, knockdown of UCHL1 also had a detrimental effect on all phenotypes, leading the authors to conclude that increases in UCHL1 represent a compensatory response to disrupted ubiquitin homeostasis.^50^

UCHL1 has also been implicated in regulating the polarization of macrophages and microglia toward a pro‐inflammatory state with an effect on production of key cytokines interleukin (IL)‐1β, IL‐6, and tumor necrosis factor α.^40^, ^41^ Although we did not find other known inflammatory proteins (such as chitinases) to be significantly raised in asymptomatic *C9orf72* carriers, it is possible that UCHL1 may represent an early marker of microglial activation.

It is, therefore, plausible that elevated UCHL1 might indicate underlying compensatory cell‐autonomous or non‐autonomous mechanisms that permit the nervous system to tolerate the deleterious effects of *C9orf72* HRE. Alternatively, it could reflect earlier neurodegenerative processes occurring upstream of the phase of accelerated neuronal loss that is associated with elevated NFL.

## Conclusions

Elevation of CSF UCHL1 occurs in asymptomatic carriers of *C9orf72* HRE before the development of sustained increases in NFL level. Longitudinal studies are needed to delineate the time course of UCHL1 elevation further, and to establish its utility in predicting genetic penetrance and timing of symptomatic disease.

## Author Contributions

A.G.T., J.D.R., K.T., and M.R.T. contributed to the conception and design of the study; E.R.D., D.G.L., B.A., I.V., R.F., E.C.E., K.Y., A.S.E., T.D., K.T., J.D.R., A.G.T., and M.R.T. contributed toward acquisition and analysis of data; E.R.D. and A.G.T. contributed to drafting of text and preparation of figures.

## Potential Conflicts of Interest

Nothing to report.

## Supporting information


**Table S1.** Participant demographics by pairwise contrast.
**Figure S1.** Quality control of proteomics dataset.
**Figure S2.** Analysis of pre‐selected target proteins in disease groups compared to age‐matched controls.
**Figure S3.** NFL and UCHL1 in cohort groups.
**Figure S4.** NFL and UCHL1 associations with age after exclusion of outlying sample.
**Figure S5.** Unbiased CSF proteomic analysis in disease groups compared to age‐matched controls.
**Figure S6.** Unbiased CSF proteomic analysis in disease groups compared to age‐matched controls without normalisation of proteomic data.
**Figure S7.** Unbiased CSF proteomic analysis in asymptomatic *C9orf72* HRE carriers without normalisation of proteomic data.

## Data Availability

The mass spectrometry proteomics data have been deposited to the ProteomeXchange Consortium via the PRIDE partner repository (https://proteomecentral.proteomexchange.org/cgi/GetDataset) with the dataset identifier PXD051859.^51^
