# Supplementary material for: Elevated Cerebrospinal Fluid Ubiquitin Carboxyl‐Terminal Hydrolase Isozyme L1 in Asymptomatic C9orf72 Hexanucleotide Repeat Expansion Carriers
Source: Ann Neurol. 2024 Nov 16;97(3):449–59. doi: 10.1002/ana.27133 (PMC11831881; doi:10.1002/ana.27133)
Supplement: Supplementary file 1 — Table S1. Participant demographics by pairwise contrast. Figure S1. Quality control of proteomics dataset. Figure S2. Analysis of pre‐selected target proteins in disease groups compared to age‐matched controls. Figure S3. NFL and UCHL1 in cohort groups. Figure S4. NFL and UCHL1 associations with age after exclusion of outlying sample. Figure S5. Unbiased CSF proteomic analysis in disease groups compared to age‐matched controls. Figure S6. Unbiased CSF proteomic analysis in disease groups compared to age‐matched controls without normalisation of proteomic data. Figure S7. Unbiased CSF proteomic analysis in asymptomatic C9orf72 HRE carriers without normalisation of proteomic data. [file ANA-97-449-s001.docx]

**Supplemental Online Content**

**Cerebrospinal fluid Ubiquitin carboxyl-terminal hydrolase isozyme L1 is elevated in asymptomatic C9orf72 hexanucleotide repeat expansion carriers**

**Table S1.** Participant demographics by pairwise contrast

**Figure S1.** Quality control of proteomics dataset.

**Figure S2.** Analysis of pre-selected target proteins in disease groups compared to age-matched controls.

**Figure S3**. NFL and UCHL1 in cohort groups

**Figure S4.** NFL and UCHL1 associations with age after exclusion of outlying sample

**Figure S5**. Unbiased CSF proteomic analysis in disease groups compared to age-matched controls.

**Figure S6.** Unbiased CSF proteomic analysis in disease groups compared to age-matched controls without normalisation of proteomic data.

**Figure S7.** Unbiased CSF proteomic analysis in asymptomatic *C9orf72* HRE carriers without normalisation of proteomic data.

=
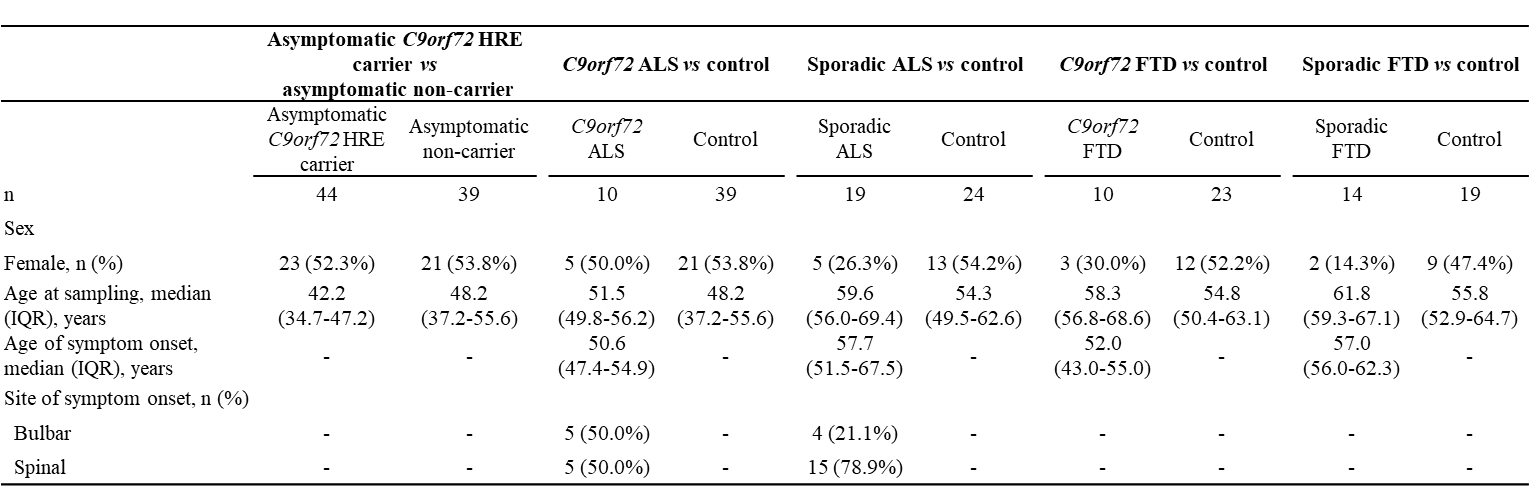

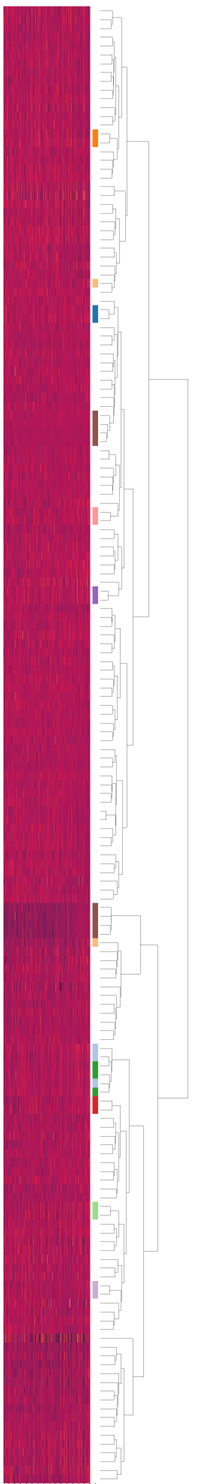

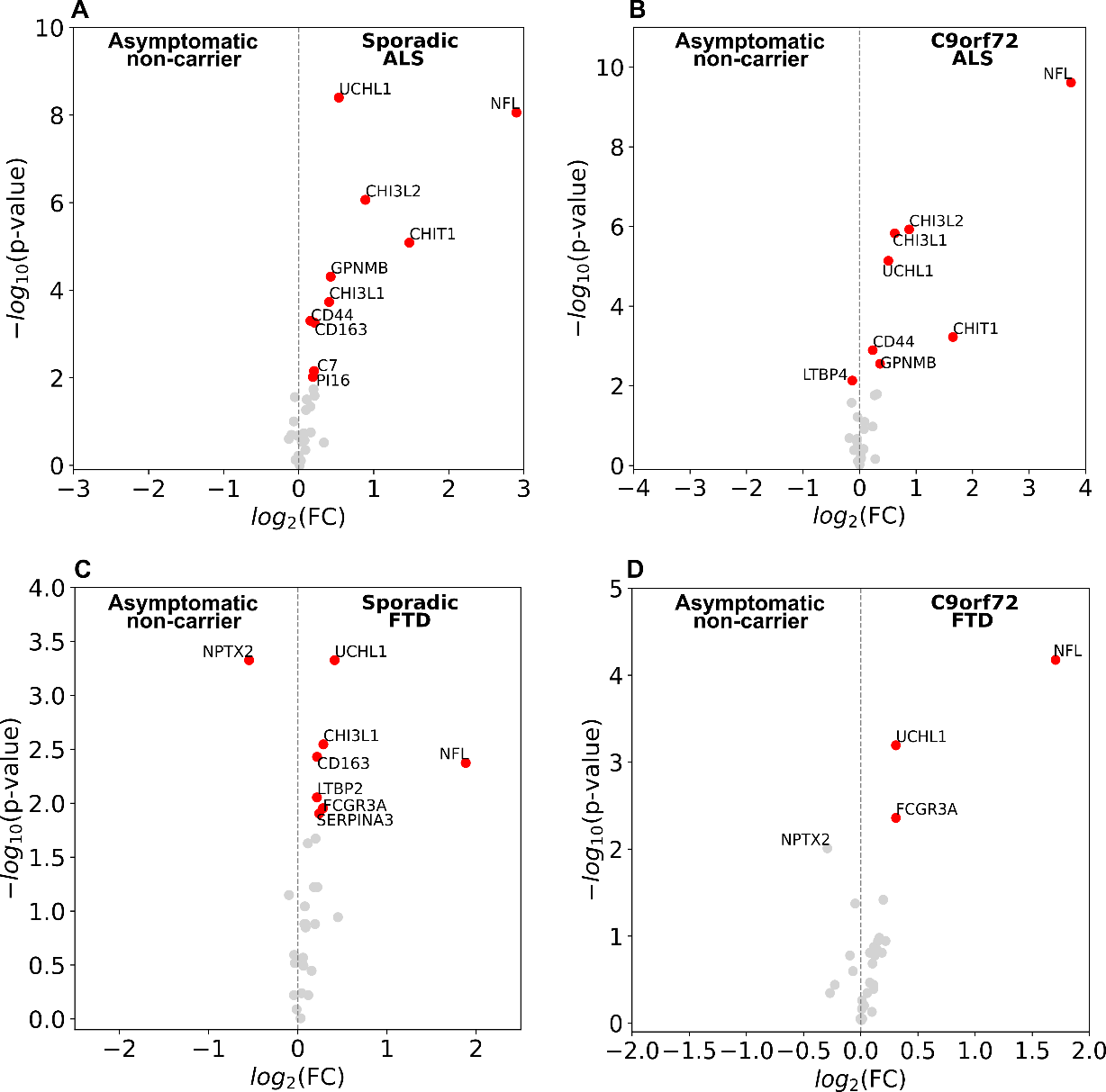

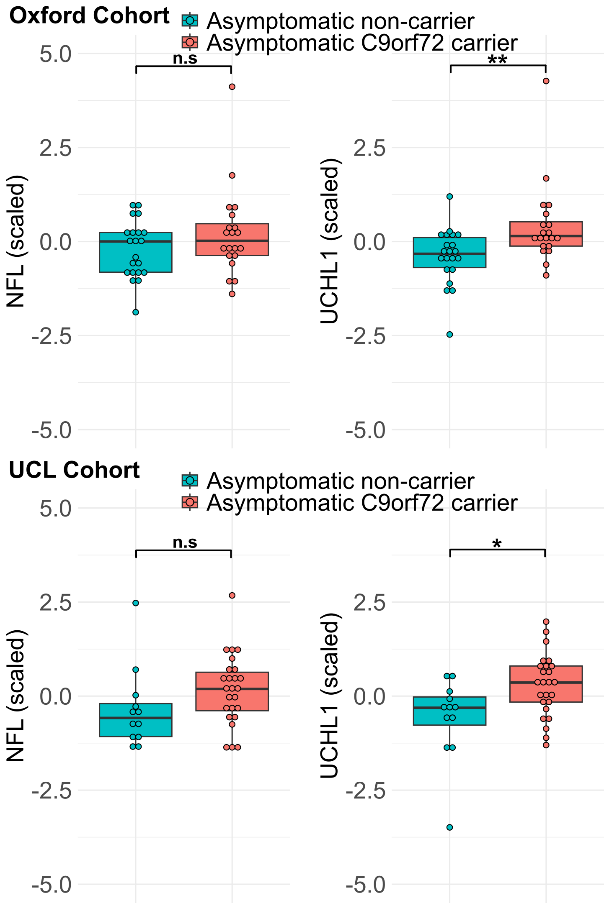


**Table S1. Participant demographics by pairwise contrast**

ALS – amyotrophic lateral sclerosis; ALSFRS-R – revised ALS functional rating scale; FTD – frontotemporal dementia; IQR – interquartile range; SD – standard deviation.

**Figure S1. Quality control of proteomics dataset.**

Hierarchical clustering of CSF samples by all proteins in mass spectrometry dataset. Colour bars represent samples prepared in duplicate (independent tryptic digests) for quality control purposes or pool created from digested peptides (brown). Heatmap demonstrates known duplicates cluster well, with samples clustering more closely than known duplicates excluded from further analysis.

**Figure S2: Analysis of pre-selected target proteins in disease groups compared to age-matched controls.**

Volcano plots showing log2 fold change versus raw p-value for **A**. Sporadic ALS **B** C9orf72 ALS, **C** Sporadic FTD **D** C9orf72 FTD compared to age-matched (p>0.1) non-carrier controls for preselected target proteins. Red points indicate proteins reaching statistical significance at FDR <0.05. NFL measured by electro chemiluminescent assay, all other proteins quantified by mass spectrometry. Significance tested by Wilcoxon Rank Sum test with FDR correction by Benjamini Hochberg procedure.

**Figure S3. NFL and UCHL1 in cohort groups**

Boxplots showing NFL and UCHL levels for asymptomatic C9orf72 HRE carriers (n=25) and non-carrier controls (n=12) in the UCL cohort, and for asymptomatic C9orf72 HRE carriers (n=20) and non-carrier controls (n=21) in the Oxford cohort. Values log-transformed, centred to zero and scaled to unit variance for comparability of effect sizes. NFL measured by electro chemiluminescent assay, UCHL1 quantified by mass spectrometry. NFL – Neurofilament light chain. UCHL1 – Ubiquitin carboxyl-terminal hydrolase isozyme L1. Significance tested by Wilcoxon Rank Sum * p < 0.05, ** p <0.01, *** p <0.001, **** p <0.0001.


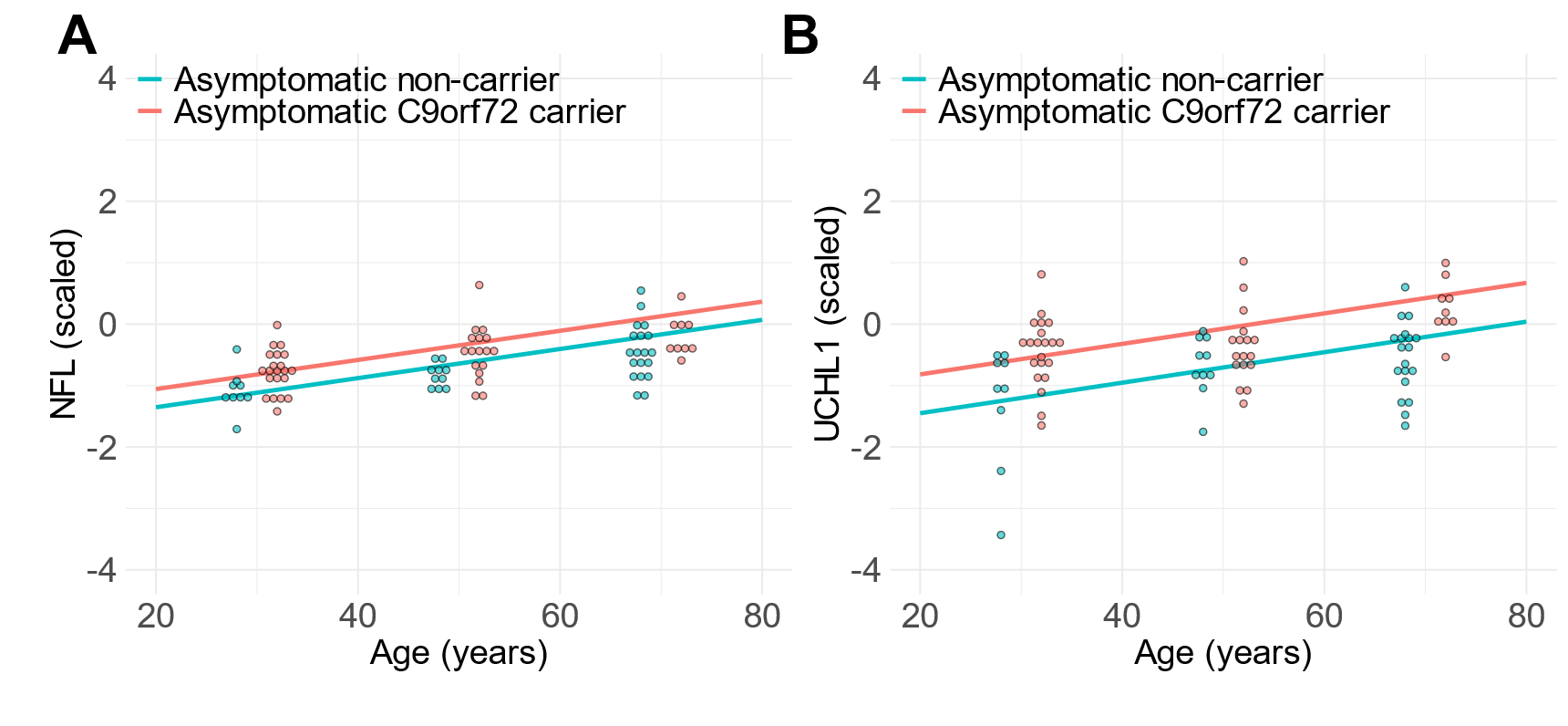


**Figure S4. NFL and UCHL1 associations with age after exclusion of outlying sample**

**A** Regression of NFL level against age in asymptomatic *C9orf72* HRE carriers and non-carrier controls. **B** Regression of UCHL1 level against age in asymptomatic *C9orf72* HRE carriers and non-carrier controls. Overlaid points binned by age (<37 years, 38-48 years, >48 years). Mean age within these age groupings did not differ significantly (p=0.47, p=0.78 and p=0.41 respectively). Values log-transformed, centred to zero and scaled to unit variance for comparability of effect sizes. NFL – Neurofilament light chain. UCHL1 – Ubiquitin carboxyl-terminal hydrolase isozyme L1.

**
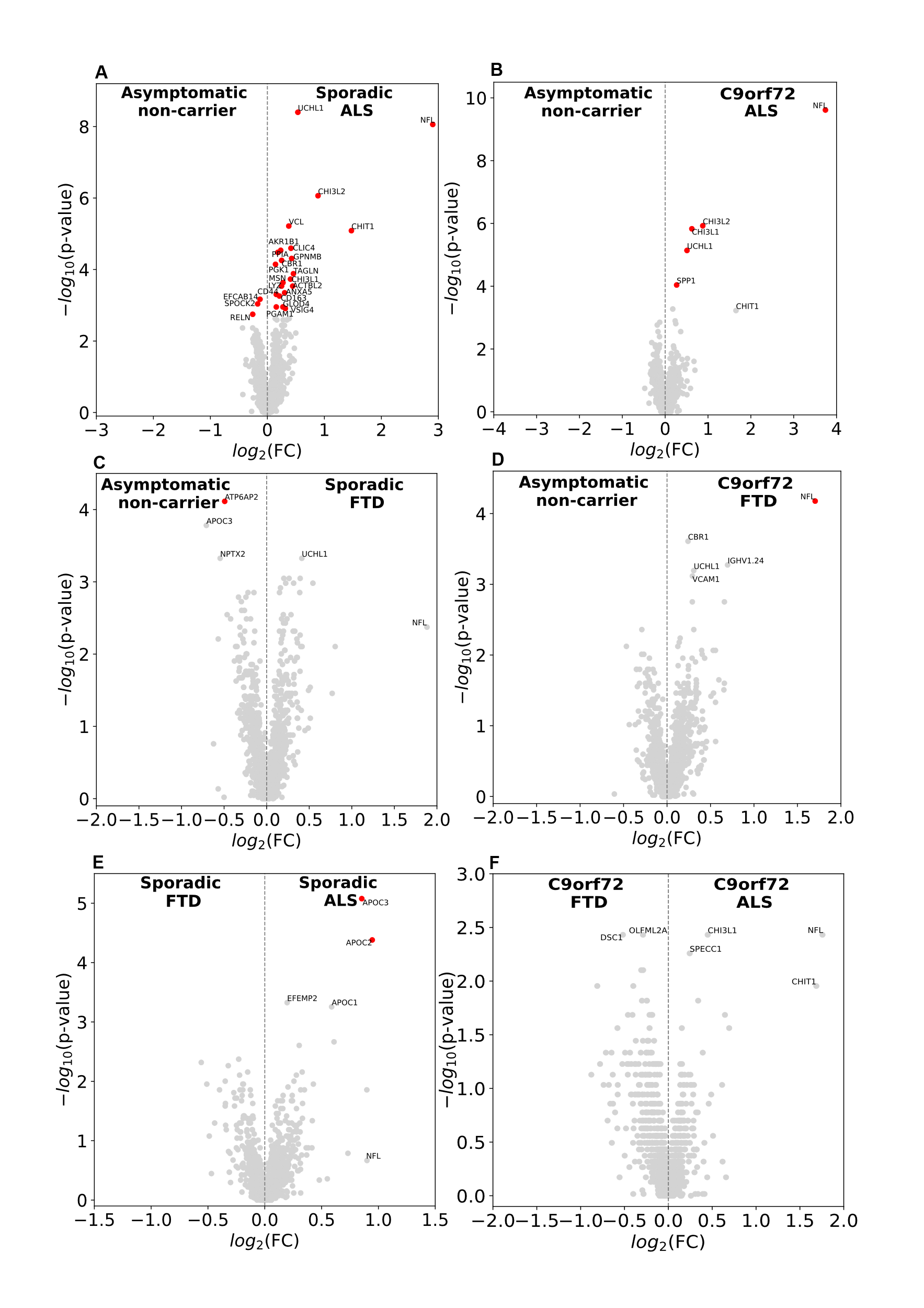
**

**Figure S5. Unbiased CSF proteomic analysis in disease groups compared to age-matched controls.**

Volcano plots showing log2 fold change versus raw p-value for **A**. Sporadic ALS **B** C9orf72 ALS, **C** Sporadic FTD **D** C9orf72 FTD compared to age-matched (p>0.1) non-carrier controls. **E** Sporadic ALS versus age-matched sporadic FTD and **F** C9orf72 ALS versus age-matched C9orf72 FTD. Red points indicate proteins reaching statistical significance at FDR <0.1. NFL measured by electro chemiluminescent assay, all other proteins quantified by mass spectrometry. Significance tested by Wilcoxon Rank Sum test with FDR correction by Benjamini Hochberg procedure.

**
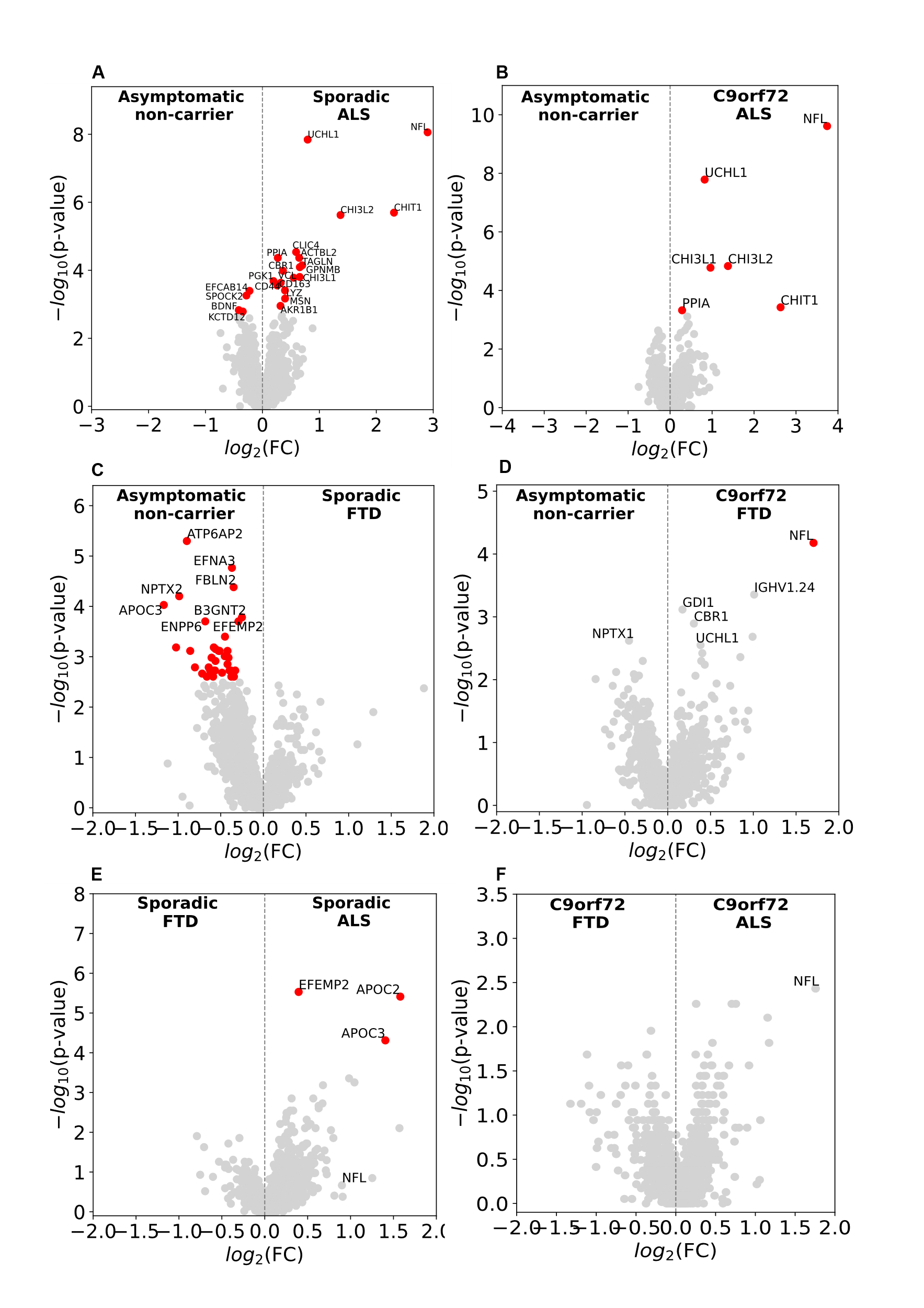
**

**Figure S6. Unbiased CSF proteomic analysis in disease groups compared to age-matched controls without normalisation of proteomic data.**

Volcano plots showing log2 fold change versus raw p-value for **A**. Sporadic ALS **B** C9orf72 ALS, **C** Sporadic FTD **D** C9orf72 FTD compared to age-matched (p>0.1) non-carrier controls. **E** Sporadic ALS versus age-matched sporadic FTD and **F** C9orf72 ALS versus age-matched C9orf72 FTD. Red points indicate proteins reaching statistical significance at FDR <0.1. NFL measured by electro chemiluminescent assay, all other proteins quantified by mass spectrometry. Significance tested by Wilcoxon Rank Sum test with FDR correction by Benjamini Hochberg procedure.

**
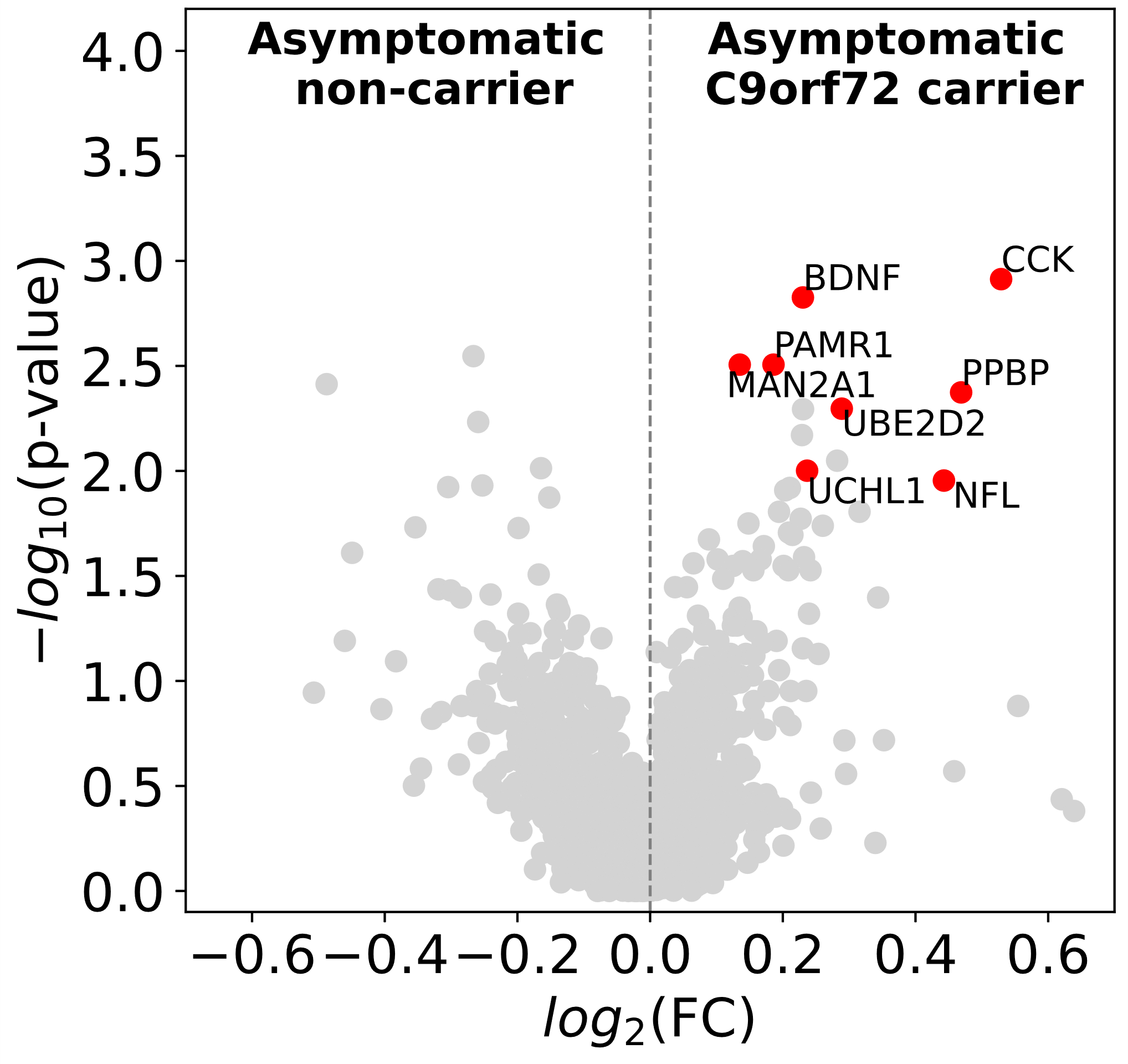
**

**Figure S7. Unbiased CSF proteomic analysis in asymptomatic *C9orf72* HRE carriers without normalisation of proteomic data.**

Volcano plots showing log2 fold change versus raw p-value for asymptomatic *C9orf72* HRE carrier**s** compared to age-matched (p>0.1) non-carrier controls. Red points indicate proteins with FDR lower than UCHL1 in normalised analysis. NFL measured by electro chemiluminescent assay, all other proteins quantified by mass spectrometry. Significance tested by Wilcoxon Rank Sum test with FDR correction by Benjamini Hochberg procedure.
